# Relation between Mass Sensitivity and Complex Power Flow in Love Wave Sensors

**DOI:** 10.3390/s22166100

**Published:** 2022-08-15

**Authors:** Piotr Kiełczyński

**Affiliations:** Institute of Fundamental Technological Research, Polish Academy of Sciences, ul. Pawińskiego 5B, 02-106 Warsaw, Poland; pkielczy@ippt.pan.pl; Tel.: +48-(22)-826-1281 (ext. 416); Fax: +48-(22)-826-9815

**Keywords:** Love wave sensors, active and reactive power flow, Poynting vector, mass sensitivity

## Abstract

In this paper, we investigate the connection between average power flows in Love wave waveguides with the mass sensitivity of Love wave sensors. In fact, loading with a Newtonian liquid gives rise to two extra power flows, in the transverse direction towards the loading Newtonian liquid. The first is an active power flow feeding viscous losses in the Newtonian liquid and the second is a reactive power flow that is responsible for the phase delay of the Love wave and consequently for the changes in phase velocity of the Love wave. Since loading with a lossless mass also leads to changes in the phase velocity, we assert that mass sensitivity $S_{\sigma }^{v_{p}}$ of Love wave sensors is connected to the average reactive power flow, in the transverse direction $x_{2}$, bouncing back and forth, between the interior of the waveguide and the loading Newtonian liquid. Subsequently, we found the thickness of the effective surface layer of mass that is equivalent to loading with a semi-infinite Newtonian liquid. The analytical formulas developed in this paper are illustrated by the results of numerical calculations performed for an exemplary Love wave waveguide composed of a PMMA surface layer deposited on an ST-Quartz substrate.

## 1. Introduction

Bulk and surface elastic waves [1,2,3,4] are widely used in sensors of physical properties of materials as well as in biosensors and chemosensors [5,6,7,8,9,10,11,12,13,14]. The elastic surface waves of the Love type are especially attractive for use in sensors working in a liquid environment, since Love surface waves can propagate long distances when loaded by low viscosity liquids, such as water. Since most chemosensors and biosensors [15,16,17,18] are used in a water-like environment, the Love wave sensors offer a significant advantage over other types of elastic sensors based on bulk elastic waves or Rayleigh-type elastic surface waves.

In this paper, we will try to connect the average power flow occurring in Love wave waveguides loaded with a Newtonian liquid with some engineering parameters of Love wave sensors, such as their coefficient of mass sensitivity $S_{\sigma }^{v_{p}}$. At first glance, the connection between the average power flow and the coefficient $S_{\sigma }^{v_{p}}$ is not obvious. However, the results obtained in this paper suggest that in fact the average power flow and the mass sensitivity of the sensor are intimately related, i.e., they display the same qualitative dependencies (maxima) as a function of wave frequency f and thickness $h_{1}$ of the surface layer of the waveguide.

In Love wave waveguides, loaded with a Newtonian liquid, the average (total) power flows $P_{1}=\int_{-\infty }^{\infty }P_{1}\left(x_{2}\right)dx_{2}\;\mathrm{and}\;P_{2}=\int_{-\infty }^{\infty }P_{2}\left(x_{2}\right)dx_{2}$ in both directions, i.e., in the direction of propagation $x_{1}$ and in the transverse direction $x_{2}$, can be of the active or reactive type. The corresponding Poynting vectors $P_{1}\left(x_{2}\right)$ and $P_{2}\left(x_{2}\right)$, were defined in (Auld, 1990). In this paper we will focus on the average power flow $P_{2}$ in the transverse direction $x_{2}$ as well as on the corresponding complex Poynting vector $P_{2}\left(x_{2}\right)$ evaluated at the interface $x_{2}=0$, between the loading Newtonian liquid and the elastic surface layer of the waveguide. Indeed, we will argue that the reactive part $ImP_{2}$ of the average power flow in the transverse direction $x_{2}$ is related to mass sensitivity $S_{\sigma }^{v_{p}}$ of the Love wave sensor, whereas the active part $ReP_{2}$ of the average power flow in the transverse direction $x_{2}$ is connected to the attenuation of the Love wave.

In other words, the active power flow $ReP_{2}$ in the transverse directions $x_{2}$ feeds viscous losses in the loading Newtonian liquid and is ultimately dissipated to heat. On the other hand, the reactive power flow $ImP_{2}$ in the transverse directions $x_{2}$ gives rise to delay of the Love wave and therefore contributes to changes in its phase velocity.

The key parameter of the Love wave sensor is its coefficient of mass sensitivity $S_{\sigma }^{v_{p}}$, i.e., the relative change $\Delta v_{p}/v_{p}$ in the phase velocity $v_{p}$ due to the loading of the waveguide with the surface mass density σ $\left[\mathrm{kg}/m^{2}\right]$ [5]. The mass sensitivity $S_{\sigma }^{v_{p}}$ depends on the surface layer thickness $h_{1}$, frequency f, and other material parameters of the Love wave waveguide [19,20].

Despite the fact that the coefficient of mass sensitivity $S_{\sigma }^{v_{p}}$ was initially defined for waveguides loaded with an infinitesimally thin layer of lossless mass, in this paper we extend the notion of the mass sensitivity $S_{\sigma }^{v_{p}}$ on waveguides loaded with a semi-infinite lossy Newtonian liquid of a density $\rho_{0}$ and viscosity $\eta_{0}$. To this end, we introduce the notion of the effective layer of thickness $h^{\prime }=\delta_{0}/2$ that provides the same phase delay as a semi-infinite Newtonian liquid loading the waveguide, where $\delta_{0}$ is the penetration depth of the Love wave into the Newtonian liquid. As a result, the equivalent surface mass density σ of a semi-infinite Newtonian liquid loading Love wave waveguide equals $\sigma =\rho_{0}\left(\delta_{0}/2\right)$ [$\mathrm{kg}/m^{2}]$, see Section 4.2.

The analytical formulas for the mass sensitivity $S_{\sigma }^{v_{p}}$ and the reactive power flow $ImP_{2}$ in the transverse direction $x_{2}$, were supported by the numerical calculations performed for a Love wave waveguide composed of a PMMA-Poly(methyl methacrylate) surface layer deposited on ST-Quartz substrate. In fact, Quartz is very attractive as a material for substrates in Love wave sensors, since it is the only common piezoelectric material that supports pure shear SH bulk waves that can be generated and received via a piezoelectric effect, using interdigital transducers in a wide range of frequencies, namely from ~1 MHz to ~1 GHz. On the other hand, PMMA is a very good candidate as a material for surface layers in Love wave waveguides, due to its low phase velocity (~1100 m/s) of bulk SH waves that promotes strong energy trapping in the surface layer itself.

To the best of our knowledge, the relationship between the mass sensitivity $S_{\sigma }^{v_{p}}$ and complex power flow $P_{2}$ in Love wave sensors was not yet analyzed in the literature and therefore can be considered as an original contribution to the state-of-the art.

The results of the theoretical analysis and numerical calculations presented in this paper provide a deeper insight into the physical phenomena occurring in Love wave sensors and can serve as a basis for better design and optimization of Love wave sensors, biosensors, and chemosensors, working in a liquid environment.

## 2. Physical Model

**Figure 1:**
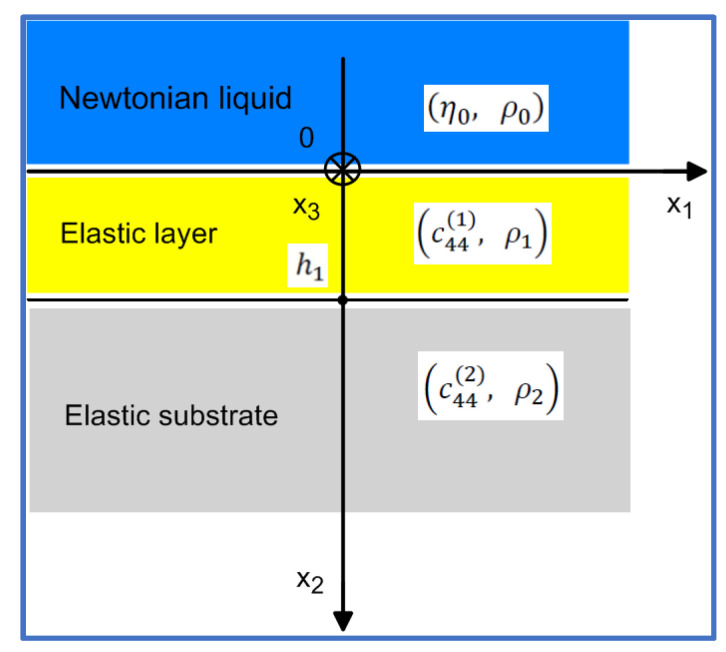
Cross-section of the waveguide employed in Love wave sensors. Elastic surface layer (PMMA) of thickness $h_{1}$ is bonded rigidly to the elastic substrate (ST-cut-Quartz). Top surface of the waveguide is in contact with a semi-infinite Newtonian liquid.

In this paper, we investigate Love wave waveguides with a single elastic surface layer rigidly bonded to a semi-infinite elastic substrate. The top surface of the waveguide is loaded with a Newtonian liquid of a semi-infinite extent (see Figure 1).

The waveguide structure shown in Figure 1 represents the main components of the simplest Love wave sensor working in a liquid environment.

Part of the energy of the Love wave penetrates into the Newtonian liquid, where it is dissipated into heat. Therefore, wavenumber k of the Love wave is a complex quantity given by the following formula:

(1) $$k=k_{p}+j\alpha$$

where $k_{p}=\omega /v_{p}$ determines the phase velocity $v_{p}$ of the Love wave, α is the attenuation, ω stands for the angular frequency and “j” is the imaginary unit.

## 3. Mathematical Model

Love surface waves, analyzed in this paper, are time-harmonic, propagate in the direction $x_{1}$, and are uniform along the transverse direction $x_{3}$. Therefore, their mechanical displacement $u_{3}^{\left(i\right)}$ and shear stresses $\tau_{23}^{\left(i\right)}$ in the Newtonian liquid (i=0), elastic surface layer (i=1) and in the elastic substrate (i=2), see Figure 1, will be sought in the following generic form:

(2a) $$u_{3}^{\left(i\right)}=u_{3}^{\left(i\right)}\left(x_{2}\right)exp\left[j\left(k\cdot x_{1}-\omega t\right)\right]$$

(2b) $$\tau_{23}^{\left(i\right)}=\tau_{23}^{\left(i\right)}\left(x_{2}\right)exp\left[j\left(k\cdot x_{1}-\omega t\right)\right]$$

(2c) $$\tau_{13}^{\left(i\right)}=\tau_{13}^{\left(i\right)}\left(x_{2}\right)exp\left[j\left(k\cdot x_{1}-\omega t\right)\right]$$

where $u_{3}^{\left(i\right)}\left(x_{2}\right)$, $\tau_{23}^{\left(i\right)}$, and $\tau_{13}^{\left(i\right)}$ express variations of the mechanical displacement and shear stress in the transverse direction $x_{2}$, k is the wavenumber of the Love surface wave, ω its angular frequency and index i=0, 1 and 2.

By definition, shear stresses $\tau_{23}^{\left(i\right)}$, $\tau_{13}^{\left(i\right)}$ of the Love wave are connected to the mechanical displacement $u_{3}^{\left(i\right)}$ by the following formulas:

(3a) $$\tau_{23}^{\left(i\right)}=c_{44}^{\left(i\right)}\frac{\partial u_{3}^{\left(i\right)}}{\partial x_{2}}\;$$

(3b) $$\tau_{13}^{\left(i\right)}=c_{44}^{\left(i\right)}\frac{\partial u_{3}^{\left(i\right)}}{\partial x_{1}}$$

where $c_{44}^{\left(i\right)}$ is the shear modulus of elasticity of the constituting medium number i.

### 3.1. Differential Equations

Mechanical displacement $u_{3}^{\left(i\right)}$ of Love surface waves is governed by the following wave equation [21] resulting from the second Newton’s law of motion:

(4) $$\frac{1}{v_{i}^{2}}\frac{\partial^{2}u_{3}^{\left(i\right)}}{\partial t^{2}}=\left(\frac{\partial^{2}u_{3}^{\left(i\right)}}{\partial x_{1}^{2}}+\frac{\partial^{2}u_{3}^{\left(i\right)}}{\partial x_{2}^{2}}\right)$$

where $v_{i}={\left(c_{44}^{\left(i\right)}/\rho_{i}\right)}^{1/2}$ is the phase velocity of SH bulk waves in medium number i, $c_{44}^{\left(i\right)}$ is its shear modulus of elasticity and $\rho_{i}$ is the density.

Substituting Equation (2) for the mechanical displacement $u_{3}^{\left(i\right)}$ into wave equation Equation (4) one obtains the following ordinary differential equation of the Helmholtz type:

(5) $$\frac{d^{2}u_{3}^{\left(i\right)}\left(x_{2}\right)}{dx_{2}^{2}}+\left(k_{i}^{2}-k^{2}\right)u_{3}^{\left(i\right)}\left(x_{2}\right)=0$$

where $k_{i}=\omega /v_{i}$ is the wavenumber of bulk SH waves in medium number i=0,1 and 2.

### 3.2. Mechanical Displacement $u_{3}^{\left(i\right)}\left(x_{2}\right)$ and Shear Stresses $\tau_{23}^{\left(i\right)}\left(x_{2}\right)$, $\tau_{13}^{\left(i\right)}\left(x_{2}\right)$

#### 3.2.1. Newtonian Liquid $\left(x_{2}<0\right)$

Since the mechanical displacement $u_{3}^{\left(0\right)}\left(x_{2}\right)$ of the Love wave must vanish for $x_{2}\to -\infty$, we will seek the solution to Equation (5) in the following form:

(6) $$u_{3}^{\left(0\right)}\left(x_{2}\right)=C_{0}\cdot exp\left(q_{0}\cdot x_{2}\right)$$

where $q_{0}={\left(k^{2}-k_{0}^{2}\right)}^{1/2}$ is the complex transverse wave number of the Love wave in the Newtonian liquid and $C_{0}$ is an arbitrary constant. In order to fulfill the condition $u_{3}^{\left(0\right)}\left(x_{2}\right)\;\to 0$ for $x_{2}\to -\infty$ the real part of the transverse wavenumber $q_{0}$ must be positive, $Req_{0}>0$.

By virtue of Equation (3a), shear stress $\tau_{23}^{\left(0\right)}$ of the Love wave in the Newtonian liquid is given by:

(7) $$\tau_{23}^{\left(0\right)}=C_{0}\cdot c_{44}^{\left(0\right)}\cdot q_{0}\cdot exp\left(q_{0}\cdot x_{2}\right)\cdot exp\left[j\left(kx_{1}-\omega t\right)\right]$$

where $c_{44}^{\left(0\right)}=-j\omega \eta_{0}$ is the shear modulus of elasticity of the Newtonian liquid and $\eta_{0}$ its viscosity.

The shear stress $\tau_{23}^{\left(0\right)}$ will enter into the appropriate boundary conditions at the interface $x_{2}=0$ of the waveguide as well as into equations for the Poynting vector $P_{2}^{\left(0\right)}\left(x_{2}\right)$ of the Love wave in the transverse direction $x_{2}$.

Similarly, using Equation (3b), shear stress $\tau_{13}^{\left(0\right)}$ of the Love wave in the Newtonian liquid is given by:

(8) $$\tau_{13}^{\left(0\right)}=jkC_{0}\cdot c_{44}^{\left(0\right)}\cdot exp\left(q_{0}\cdot x_{2}\right)\cdot exp\left[j\left(kx_{1}-\omega t\right)\right]$$

where $c_{44}^{\left(0\right)}=-j\omega \eta_{0}$ is the shear modulus of elasticity of the Newtonian liquid and $\eta_{0}$ its viscosity.

The shear stress $\tau_{13}^{\left(0\right)}$ will enter into equations for the Poynting vector $P_{1}^{\left(0\right)}\left(x_{2}\right)$ of the Love wave in the direction of propagation $x_{1}$.

#### 3.2.2. Elastic Surface Layer $\left(0<x_{2}<h_{1}\right)$

Since the elastic surface layer is of a finite thickness $h_{1}$ the solution to Equation (5) can be sought in the following form:

(9) $$u_{3}^{\left(1\right)}\left(x_{2}\right)=C_{1}\cdot \sin\left(q_{1}\cdot x_{2}\right)+C_{2}\cdot \cos\left(q_{1}\cdot x_{2}\right)$$

where $C_{1}\;\mathrm{and}\;C_{2}$ are arbitrary constants and the transverse wave number of the Love wave in the elastic surface layer $q_{1}={\left(k_{1}^{2}-k^{2}\right)}^{1/2}$. Since the elastic surface layer is of a finite thickness $h_{1}$, the real part of $q_{1}$ can be either positive or negative.

By virtue of Equation (3a), shear stress $\tau_{23}^{\left(1\right)}$ of the Love wave in the elastic surface layer is given by:

(10) $$\tau_{23}^{\left(1\right)}=c_{44}^{\left(1\right)}q_{1}\left[C_{1}cos\left(q_{1}x_{2}\right)-C_{2}\sin\left(q_{1}x_{2}\right)\right]exp\left[j\left(kx_{1}-\omega t\right)\right]$$

The shear stress $\tau_{23}^{\left(1\right)}$ will enter into the appropriate boundary conditions at two interfaces $x_{2}=0$ and $x_{2}=h_{1}$ of the waveguide as well as into equations for the Poynting vector $P_{2}^{\left(1\right)}\left(x_{2}\right)$ of the Love wave in the transverse direction $x_{2}$.

Analogously, employing Equation (3b), shear stress $\tau_{13}^{\left(1\right)}$ of the Love wave in the elastic surface layer is given by:

(11) $$\tau_{13}^{\left(1\right)}=jkc_{44}^{\left(1\right)}\left[C_{1}\cdot \sin\left(q_{1}\cdot x_{2}\right)+C_{2}\cdot \cos\left(q_{1}\cdot x_{2}\right)\right]exp\left[j\left(kx_{1}-\omega t\right)\right]$$

The shear stress $\tau_{13}^{\left(1\right)}$. will enter into equations for the Poynting vector $P_{1}^{\left(1\right)}\left(x_{2}\right)$ of the Love wave in the direction of propagation $x_{1}$.

#### 3.2.3. Elastic Substrate $\left(x_{2}>h_{1}\right)$

Since the amplitude of the Love surface wave in the elastic substrate must tend to zero for $x_{2}\to \infty$, as a solution to Equation (5) we choose the following expression:

(12) $$u_{3}^{\left(2\right)}\left(x_{2}\right)=C_{3}\cdot exp\left(-q_{2}\cdot x_{2}\right)$$

where the transverse wave number of the Love wave in the substrate $q_{2}={\left(k^{2}-k_{2}^{2}\right)}^{1/2}$ must fulfill the condition $Re\left(q_{2}\right)>0$. Moreover, $k_{2}=\omega /v_{2}$ is the wavenumber of SH bulk waves in the substrate and $v_{2}={\left(c_{44}^{\left(2\right)}/\rho_{2}\right)}^{1/2}$ is the phase velocity of bulk SH waves in the substrate, $c_{44}^{\left(2\right)}$ is its modulus of elasticity and $\rho_{2}$ is its density.

By virtue of Equation (3a), shear stress $\tau_{23}^{\left(2\right)}$ of the Love wave in the elastic substrate is given by:

(13) $$\tau_{23}^{\left(2\right)}=-C_{3}\cdot c_{44}^{\left(2\right)}\cdot q_{2}\cdot exp\left(-q_{2}\cdot x_{2}\right)\cdot exp\left[j\left(kx_{1}-\omega t\right)\right]$$

The shear stress $\tau_{23}^{\left(2\right)}$ will enter into the appropriate boundary conditions at the interface $x_{2}=h_{1}$ of the waveguide as well as into equations for the Poynting vector $P_{2}^{\left(2\right)}\left(x_{2}\right)$ of the Love wave in the transverse direction $x_{2}$.

On the other hand, using Equation (3b), shear stress $\tau_{13}^{\left(2\right)}$ of the Love wave in the elastic substrate is given by:

(14) $$\tau_{13}^{\left(2\right)}=jkC_{3}\cdot c_{44}^{\left(2\right)}\cdot exp\left(-q_{2}\cdot x_{2}\right)\cdot exp\left[j\left(kx_{1}-\omega t\right)\right]$$

The shear stress $\tau_{13}^{\left(2\right)}$ will enter into equations for the Poynting vector $P_{1}^{\left(2\right)}\left(x_{2}\right)$ of the Love wave in the direction of propagation $x_{1}$.

### 3.3. Boundary Conditions

Boundary conditions at two interfaces $x_{2}=0$ and $x_{2}=h_{1}$ of the waveguide shown in Figure 1 require the continuity of the mechanical displacement $u_{3}^{\left(i\right)}$ and shear stress $\tau_{23}^{\left(i\right)}$ [1]. Consequently, at the interface $x_{2}=0$ between the Newtonian liquid, and the elastic surface layer we can write:

(15) $${\left.u_{3}^{\left(0\right)}\right|}_{x_{2}=0}={\left.u_{3}^{\left(1\right)}\right|}_{x_{2}=0}$$

(16) $${\left.\tau_{23}^{\left(0\right)}\right|}_{x_{2}=0}={\left.\tau_{23}^{\left(1\right)}\right|}_{x_{2}=0}$$

Similarly, at the interface $x_{2}=h_{1}$ between the elastic surface layer and the elastic substrate we have:

(17) $${\left.u_{3}^{\left(1\right)}\right|}_{x_{2}=h_{1}}={\left.u_{3}^{\left(2\right)}\right|}_{x_{2}=h_{1}}$$

(18) $${\left.\tau_{23}^{\left(1\right)}\right|}_{x_{2}=h_{1}}={\left.\tau_{23}^{\left(2\right)}\right|}_{x_{2}=h_{1}}$$

Substituting Equations (6), (7), (9), (10), (12) and (13) into boundary conditions Equations (15)–(18), one obtains the following set of homogeneous linear algebraic equations for the unknown coefficients $C_{0},\;C_{1},\;C_{2}\;\mathrm{and}\;C_{3}$:

(19) $$\left[\begin{array}{cccc} 1 & 0 & -1 & 0\\ c_{44}^{\left(0\right)}q_{0} & -c_{44}^{\left(1\right)}q_{1} & 0 & 0\\ 0 & sin\left(q_{1}h_{1}\right) & cos\left(q_{1}h_{1}\right) & -exp\left(-q_{2}h_{1}\right)\\ 0 & \left(c_{44}^{\left(1\right)}q_{1}\right)\cdot cos\left(q_{1}h_{1}\right) & -\left(c_{44}^{\left(1\right)}q_{1}\right)\cdot sin\left(q_{1}h_{1}\right) & \left(c_{44}^{\left(2\right)}q_{2}\right)\cdot exp\left(-q_{2}h_{1}\right) \end{array}\right]\left[\begin{array}{c} C_{0}\\ C_{1}\\ \begin{array}{c} C_{2}\\ C_{3} \end{array} \end{array}\right]=\left[\begin{array}{c} 0\\ 0\\ \begin{array}{c} 0\\ 0 \end{array} \end{array}\right]$$

Since the set of 4 linear algebraic equations (Equation (19)) is homogeneous we can determine only 3 independent coefficients $C_{i}$ in function of the remaining one, say $C_{0}$. Consequently, the coefficients $C_{1},\;C_{2},\;C_{3}$ can be expressed in terms of the coefficient $C_{0}$ as:

(20) $$\begin{array}{l} C_{1}=C_{0}\;\frac{c_{44}^{\left(0\right)}q_{0}}{c_{44}^{\left(1\right)}q_{1}}\\ C_{2}=C_{0}\\ C_{3}=C_{0}e^{q_{2}h_{1}}\left[\frac{c_{44}^{\left(0\right)}q_{0}}{c_{44}^{\left(1\right)}q_{1}}sin\left(q_{1}h_{1}\right)+cos\left(q_{1}h_{1}\right)\right] \end{array}$$

### 3.4. Dispersion Equation

The set of homogeneous linear algebraic equations given by Formula (19) in a matrix form has a non-trivial solution if the determinant of its left-hand matrix equals zero. This condition leads to the following dispersion relation for the phase velocity and attenuation of the Love wave propagating in the investigated waveguide shown in Figure 1:

(21) $$\left[{\left(c_{44}^{\left(1\right)}q_{1}\right)}^{2}-c_{44}^{\left(2\right)}q_{2}c_{44}^{\left(0\right)}q_{0}\right]tan\left(q_{1}h_{1}\right)-c_{44}^{\left(1\right)}q_{1}\left[c_{44}^{\left(0\right)}q_{0}+c_{44}^{\left(2\right)}q_{2}\right]=0$$

Formula (21) is a complex dispersion equation of the Love wave, propagating in the waveguide structure shown in Figure 1. Equation (21) can be split into its real and imaginary parts, providing therefore a set of two nonlinear algebraic equations for unknown $k_{p}$ and α [21]. The resulting set of two nonlinear transcendental algebraic equations can be solved numerically, using for instance a two-dimensional Newton–Raphson procedure.

Indeed, solving numerically the dispersion equation is a prerequisite in the analysis of Love surface waves, propagating in any waveguide structure, since without the knowledge of the complex wavenumber $k=k_{p}+j\alpha$, we cannot evaluate numerical values of other field quantities of the Love wave, such as the mechanical displacement $u_{3}$, shear stress $\tau_{23}$ or Poynting vector $P_{2}$.

### 3.5. Complex Poynting Vector $P_{2}\left(x_{2}\right)$, in the Transverse Direction $x_{2}$

The power flux in the investigated Love wave waveguide is represented by the complex Poynting vector with two components, i.e., $P_{1}\left(x_{2}\right)$ in the direction of propagation $x_{1}$ and $P_{2}\left(x_{2}\right)$ in the transverse direction $x_{2}$.

In this section, we will develop analytical formulas for the complex Poynting vector $P_{2}\left(x_{2}\right)$ in the transverse direction $x_{2}$ of the waveguide that is loaded with a Newtonian liquid at its surface $\left(x_{2}=0\right)$.

In free waveguides not loaded with a Newtonian liquid, the power flux $P_{2}\left(x_{2}\right)$ across the free interface $x_{2}=0$ is obviously zero, $P_{2}\left(x_{2}=0\right)=0$, since no power can be transmitted from the waveguide into vacuum.

This situation changes drastically in waveguides loaded with a Newtonian liquid, since the acoustic power flux $P_{2}\left(x_{2}\right)$ across the interface $x_{2}=0$ is now clearly non-zero, $P_{2}\left(x_{2}=0\right)\neq 0$. As a result, an additional complex power flow, between the interior of the waveguide and the bulk of the loading Newtonian liquid, occurs.

Thus, the complex Poynting vector $P_{2}\left(x_{2}\right)=ReP_{2}^{\left(0\right)}\left(x_{2}\right)+jImP_{2}^{\left(0\right)}\left(x_{2}\right)$, evaluated at the interface $x_{2}=0$, contains unique information about viscous $\eta_{0}$ and inertial $\rho_{0}$ properties of the loading Newtonian liquid.

By definition, the complex Poynting vector $P_{2}\left(x_{2}\right)\;\left[W/m^{2}\right]$ in the transverse direction $x_{2}$ can be expressed as [2]:

(22) $$P_{2}\left(x_{2}\right)=-\frac{1}{2}\tau_{23}\left(x_{2}\right){\left[-j\omega u_{3}\left(x_{2}\right)\right]}^{*}$$

where the asterisk “*” stands for complex conjugation and “j” is the imaginary unit.

Due to the interaction with the viscous Newtonian liquid, Love surface waves propagating in the direction $x_{1}$ in the waveguide structure shown in Figure 1, will be gradually attenuated, what can be expressed analytically as $P_{2}\left(x_{1},x_{2}\right)=P_{2}\left(x_{2}\right)e^{-2\alpha x_{1}}$

#### 3.5.1. Newtonian Liquid $\left(x_{2}<0\right)$

Substituting Equations (6) and (7), for the mechanical displacement $u_{3}^{\left(0\right)}\left(x_{2}\right)$ and shear stress $\tau_{23}^{\left(0\right)}\left(x_{2}\right)$ of the Love wave in the Newtonian liquid, as well as Formula (20) for the coefficients $C_{1},\;C_{2},\;C_{3}$ into Equation (22) one obtains the following expression for the complex Poynting vector $P_{2}^{\left(0\right)}\left(x_{2}\right)$ in the Newtonian liquid along, the transverse direction $x_{2}$:

(23) $$P_{2}^{\left(0\right)}\left(x_{2}\right)=-j\frac{\omega }{2}{\left|C_{0}\right|}^{2}c_{44}^{\left(0\right)}q_{0}e^{2Re\left(q_{0}\right)x_{2}}$$

where $Re\left(q_{0}\right)>0$.

#### 3.5.2. Elastic Surface Layer $\left(h_{1}>x_{2}>0\right)$

Substituting Equations (9) and (10), for the mechanical displacement $u_{3}^{\left(1\right)}\left(x_{2}\right)$ and shear stress $\tau_{23}^{\left(1\right)}\left(x_{2}\right)$ of the Love wave in the elastic surface layer, as well as Formula (20) for the coefficients $C_{1},\;C_{2},\;C_{3}$ into Equation (22), one obtains the following expression for the complex Poynting vectors $P_{2}^{\left(1\right)}\left(x_{2}\right)$ in the elastic surface, along the transverse direction $x_{2}$:

(24) $$P_{2}^{\left(1\right)}\left(x_{2}\right)=-j\frac{\omega }{2}{\left|C_{0}\right|}^{2}F_{2}\left(q_{1}x_{2}\right)F_{1}^{*}\left(q_{1}x_{2}\right)$$

where the auxiliary functions $F_{1}\left(q_{1}x_{2}\right)=$ $\left(c_{44}^{\left(0\right)}q_{0}/c_{44}^{\left(1\right)}q_{1}\right)sin\left(q_{1}x_{2}\right)-cos\left(q_{1}x_{2}\right)$ and $F_{2}\left(q_{1}x_{2}\right)=c_{44}^{\left(0\right)}q_{0}cos\left(q_{1}x_{2}\right)-c_{44}^{\left(1\right)}q_{1}sin\left(q_{1}x_{2}\right)$.

#### 3.5.3. Elastic Substrate $\left(x_{2}>h_{1}\right)$

Substituting Equations (12) and (13), for the mechanical displacement $u_{3}^{\left(2\right)}\left(x_{2}\right)$ and shear stress $\tau_{23}^{\left(2\right)}\left(x_{2}\right)$ of the Love wave in the elastic substrate, as well as Formula (20) for the coefficients $C_{1},\;C_{2},\;C_{3}$ into Equation (22), one obtains the following expression for the complex Poynting vectors $P_{2}^{\left(2\right)}\left(x_{2}\right)$ in the elastic substrate, along the transverse direction $x_{2}$:

(25) $$P_{2}^{\left(2\right)}\left(x_{2}\right)=-j\frac{\omega }{2}{\left|C_{0}\right|}^{2}F_{2}\left(q_{1}h\right)F_{1}^{*}\left(q_{1}h\right)e^{-2Re\left(q_{2}\right)\left(x_{2}-h_{1}\right)}$$

where $Re\left(q_{2}\right)>0$ and $h_{1}$ stands for thickness of the elastic surface layer.

#### 3.5.4. Complex Poynting Vector $P_{2}\left(x_{2}\right)$ Evaluated at the Interface $x_{2}=0$ between Newtonian Liquid and Elastic Surface Layer

As it was stated before in this section, the complex Poynting vector $P_{2}\left(x_{2}\right)$ evaluated at the interface $x_{2}=0$ contains unique information about viscous $\eta_{0}$ and inertial $\rho_{0}$ properties of the loading Newtonian liquid.

According to Equation (23) the complex Poynting vector $P_{2}^{\left(0\right)}\left(x_{2}\right)$ evaluated at the interface $x_{2}=0$ is given by:

(26) $$P_{2}^{\left(0\right)}\left(x_{2}=0\right)=-j\frac{\omega }{2}{\left|C_{0}\right|}^{2}c_{44}^{\left(0\right)}q_{0}$$

The transverse wavenumber $q_{0}$ of the Love wave in the Newtonian liquid is a complex quantity; therefore, it can be represented as $q_{0}=a_{0}+jb_{0}$. On the other hand, the elastic modulus of the Newtonian liquid $c_{44}^{\left(0\right)}=-j\omega \eta_{0}$. Therefore, Equation (26) can be written as:

(27) $$P_{2}^{\left(0\right)}\left(x_{2}=0\right)=-\frac{\omega^{2}}{2}{\left|C_{0}\right|}^{2}\eta_{0}a_{0}-j\frac{\omega^{2}}{2}{\left|C_{0}\right|}^{2}\eta_{0}b_{0}$$

Since for the Newtonian liquid $q_{0}={\left(k^{2}-k_{0}^{2}\right)}^{1/2}$ and $k_{0}^{2}=\omega^{2}\rho_{0}/\left(-j\omega \eta_{0}\right)$ as well as the wavenumber of the Love wave $k=k_{p}+j\alpha$, we can write the following equation:

(28) $$q_{0}^{2}=k_{p}^{2}-\alpha^{2}+j\left(k_{p}\alpha +\frac{\omega \rho_{0}}{\eta_{0}}\right)$$

Thus, real $a_{0}$ and imaginary $b_{0}$ part of the transverse wavenumber $q_{0}$ of the Love wave in the Newtonian liquid equal:

(29) $$a_{0}=\sqrt{\frac{k_{p}^{2}-\alpha^{2}}{2}\left[\sqrt{1+{\left(\frac{k_{p}\alpha +\frac{\omega \rho_{0}}{\eta_{0}}}{k_{p}^{2}-\alpha^{2}}\right)}^{2}}+1\right]}\;b_{0}=-\sqrt{\frac{k_{p}^{2}-\alpha^{2}}{2}\left[\sqrt{1+{\left(\frac{k_{p}\alpha +\frac{\omega \rho_{0}}{\eta_{0}}}{k_{p}^{2}-\alpha^{2}}\right)}^{2}}-1\right]}$$

where $k_{p}=\omega /v_{p}$

Consequently, from Equation (27) it follows that:

(30) $$ReP_{2}^{\left(0\right)}\left(x_{2}=0\right)=-\frac{\omega^{2}}{2}{\left|C_{0}\right|}^{2}\eta_{0}a_{0}\;ImP_{2}^{\left(0\right)}\left(x_{2}=0\right)=-\frac{\omega^{2}}{2}{\left|C_{0}\right|}^{2}\eta_{0}b_{0}$$

where $a_{0}$, $b_{0}$ are given by Equation (29).

Equation (30) shows that the real and imaginary parts of the complex Poynting vector $P_{2}^{\left(0\right)}\left(x_{2}\right)$, evaluated at the interface $x_{2}=0$, are involved functions of $v_{p},\;\alpha ,\;\omega ,\;\eta_{0}\;and\;\rho_{0}$. It should be noticed that $v_{p}\;and\;\alpha$ depend in turn on the material and geometrical parameters of the waveguide and Newtonian liquid.

Since in general $ReP_{2}^{\left(0\right)}\left(x_{2}=0\right)\neq 0$ and $ImP_{2}^{\left(0\right)}\left(x_{2}=0\right)\neq 0$, Equation (30) proves that in waveguides loaded with a Newtonian liquid we observe a non-zero active power flow from the waveguide interior to the loading Newtonian liquid as well as a non-zero reactive power flow bouncing back and forth between waveguide interior and the loading Newtonian liquid.

### 3.6. Complex Poynting Vector $P_{1}\left(x_{2}\right)$, in the Direction of Propagation $x_{1}$

By definition, the complex Poynting vector $P_{1}\left(x_{2}\right)\;\left[W/m^{2}\right]$ in the direction of propagation $x_{1}$ is given by [2]:

(31) $$P_{1}\left(x_{2}\right)=-\frac{1}{2}\tau_{13}\left(x_{2}\right){\left[-j\omega u_{3}\left(x_{2}\right)\right]}^{*}$$

where the asterisk “*” stands for complex conjugation and “j” is the imaginary unit.

Since shear stress $\tau_{13}^{\left(i\right)}\left(x_{2}\right)=jkc_{44}^{\left(i\right)}u_{3}^{\left(i\right)}\left(x_{2}\right)$, see Equation (2c), the complex Poynting vector $P_{1}^{\left(i\right)}\left(x_{2}\right)$ in the constituting media (i=0,1,2) of the waveguide writes:

(32) $$P_{1}^{\left(i\right)}\left(x_{2}\right)=\frac{1}{2}kc_{44}^{\left(i\right)}{\left|u_{3}^{\left(i\right)}\left(x_{2}\right)\right|}^{2}$$

where $u_{3}^{\left(0\right)}\left(x_{2}\right)$, $u_{3}^{\left(1\right)}\left(x_{2}\right)$ and $u_{3}^{\left(2\right)}\left(x_{2}\right)$ are, respectively, the mechanical displacements in the Newtonian liquid (Equation (6)), elastic surface layer (Equation (9)), and in the elastic substrate (Equation (12)). Analogously, $c_{44}^{\left(i\right)}$ correspond to the shear modulus of elasticity of the constituting medium number i=0,1,2.

### 3.7. Average (Total) Power Flow $P_{2}=\int_{-\infty }^{\infty }P_{2}\left(x_{2}\right)dx_{2}$ in the Transverse Direction $x_{2}$

The average (total) power flow $P_{2}$ in the transverse direction $x_{2}$ is defined as:

(33) $$P_{2}=\int_{-\infty }^{\infty }P_{2}\left(x_{2}\right)dx_{2}=\overset{0}{\underset{-\infty }{\int }}P_{2}^{\left(0\right)}\left(x_{2}\right)dx_{2}+\overset{h_{1}}{\underset{0}{\int }}P_{2}^{\left(1\right)}\left(x_{2}\right)dx_{2}+\overset{\infty }{\underset{h_{1}}{\int }}P_{2}^{\left(2\right)}\left(x_{2}\right)dx_{2}$$

where $P_{2}^{\left(0\right)}\left(x_{2}\right)$, $P_{2}^{\left(1\right)}\left(x_{2}\right)$ and $P_{2}^{\left(2\right)}\left(x_{2}\right)$ are, respectively, the complex Poynting vectors in the Newtonian liquid (Equation (23)), elastic surface layer (Equation (24)) and in the elastic substrate (Equation (25)).

The integrals in Equation (33) can be evaluated analytically. However, due to their excessive length and complexity they will be not reproduced here. Since Poynting vectors $P_{2}^{\left(0\right)}\left(x_{2}\right)$, $P_{2}^{\left(1\right)}\left(x_{2}\right)$ and $P_{2}^{\left(2\right)}\left(x_{2}\right)$ are complex, the average power flow $P_{2}$ in the transverse direction $x_{2}$ is a complex-valued quantity, therefore it can be written as $P_{2}=ReP_{2}+jImP_{2}$, where in general $ReP_{2}\neq 0$ and $ImP_{2}\neq 0$.

The reactive part $ImP_{2}$ of the average power flow $P_{2}$, in the transverse direction $x_{2}$, in the Newtonian liquid $P_{2}^{\left(0\right)}=\int_{-\infty }^{0}P_{2}^{\left(0\right)}\left(x_{2}\right)dx_{2},$ will be plotted in Section 5.1, as a function of frequency f and thickness of the elastic surface layer $h_{1}$.

It should be noticed that the reactive parts of the average power flow in the elastic surface layer $P_{2}^{\left(1\right)}=\int_{0}^{h_{1}}P_{2}^{\left(1\right)}\left(x_{2}\right)dx_{2}$ and in the elastic substrate $P_{2}^{\left(2\right)}=\int_{h_{1}}^{\infty }P_{2}^{\left(2\right)}\left(x_{2}\right)dx_{2}$ are always non-zero in free waveguides, not loaded with a Newtonian liquid, since they are responsible for the energy storage occurring in Love wave waveguides in the transverse direction $x_{2}$. However, the active part of the average power flow in the elastic surface layer $P_{2}^{\left(1\right)}$ and in the elastic substrate $P_{2}^{\left(2\right)}$ is always zero in lossless waveguides, not loaded with a Newtonian liquid. By contrast, the active part of the average power flow in the elastic surface layer $P_{2}^{\left(1\right)}$, in the elastic substrate $P_{2}^{\left(2\right)}$ and in the loading Newtonian liquid is always non-zero in waveguides, loaded with a Newtonian liquid.

### 3.8. Average (Total) Power Flow $P_{1}=\int_{-\infty }^{\infty }P_{1}\left(x_{2}\right)dx_{2}$ in the Direction of Propagation $x_{1}$

The average (total) power flow $P_{1}$ in the direction of propagation $x_{1}$ is defined as:

(34) $$P_{1}=\int_{-\infty }^{\infty }P_{1}\left(x_{2}\right)dx_{2}=\overset{0}{\underset{-\infty }{\int }}P_{1}^{\left(0\right)}\left(x_{2}\right)dx_{2}+\overset{h_{1}}{\underset{0}{\int }}P_{1}^{\left(1\right)}\left(x_{2}\right)dx_{2}+\overset{\infty }{\underset{h_{1}}{\int }}P_{1}^{\left(2\right)}\left(x_{2}\right)dx_{2}$$

where $P_{1}^{\left(0\right)}\left(x_{2}\right)$, $P_{1}^{\left(1\right)}\left(x_{2}\right)$ and $P_{1}^{\left(2\right)}\left(x_{2}\right)$ are, respectively, complex Poynting vectors in the Newtonian liquid, elastic surface layer and in the elastic substrate, given by Equation (32).

The integrals in Equation (34) can be evaluated analytically. However, due to their excessive length and complexity they will be not reproduced here. Since Poynting vectors $P_{1}^{\left(0\right)}\left(x_{2}\right)$, $P_{1}^{\left(1\right)}\left(x_{2}\right)$ and $P_{1}^{\left(2\right)}\left(x_{2}\right)$ are complex, the average power flow $P_{1}$ in the direction of propagation $x_{1}$ is a complex-valued quantity as well and therefore can be written as $P_{1}=ReP_{1}+jImP_{1}$, where in general $ReP_{1}\neq 0$ and $ImP_{1}\neq 0$. The active part $ReP_{1}$ of the average power flow $P_{1}$ in the direction of propagation $x_{1}$ will serve as a normalization factor in Section 5.1.

## 4. Mass Sensitivity of Love Wave Sensors for Waveguides Loaded with an Infinitesimal Layer of Lossless Mass

### 4.1. Coefficient of Mass Sensitivity $S_{\sigma }^{v_{p}}$

One of the most important parameters characterizing the quality of Love wave sensors is the coefficient of mass sensitivity. Until now, this coefficient of mass sensitivity was evaluated using the perturbation theory and/or numerical methods such as the finite element method, etc. [21,22,23,24,25,26]. In this paper, to evaluate the mass sensitivity, we used analytical methods that provide a deeper insight into the physical background of the operation of Love wave sensors [19,20].

**Figure 2:**
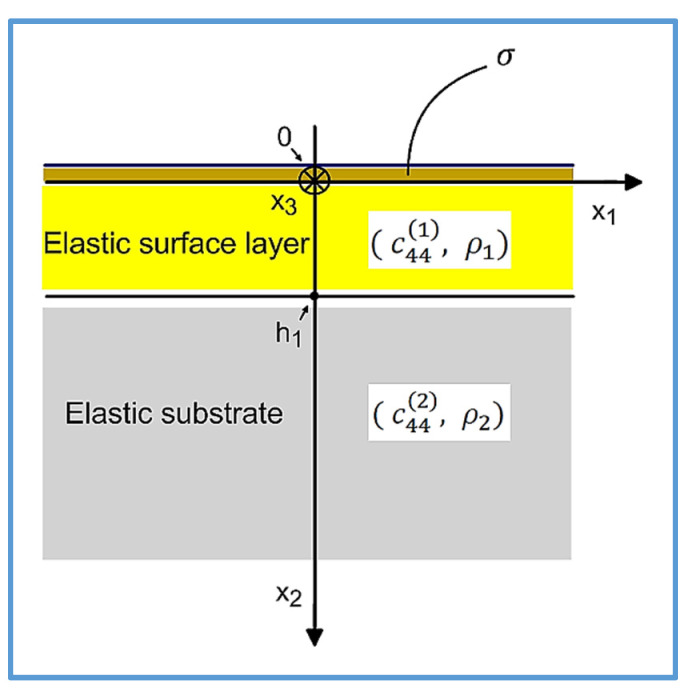
Cross-section of the Love wave waveguide loaded with an infinitesimally thin layer of lossless mass with a surface density $\sigma \;\left[\mathrm{kg}/m^{2}\right]$. Love surface waves propagate in the direction of axis $x_{1}$. Shear horizontal (SH) mechanical displacement $u_{3}$ of the Love wave is polarized along the $x_{3}$ axis.

The sensitivity of Love wave sensors to mass loading was initially defined in lossless waveguides, where a semi-infinite Newtonian liquid in Figure 1 is replaced by an infinitesimal layer of lossless mass that only changes the phase velocity $v_{p}$ of the Love wave, without introducing any extra attenuation, see Figure 2. 

The coefficient of mass sensitivity $S_{\sigma }^{v_{p}}$ of Love wave sensors is defined as:

(35) $$S_{\sigma }^{v_{p}}=\frac{1}{v_{p}}\frac{dv_{p}}{d\sigma }$$

It can be shown [20] that the dispersion equation for Love surface waves propagating in lossless waveguides loaded with an infinitesimal layer of lossless mass density σ is formally identical to Equation (21) developed in Section 3.4 in this paper, providing that the term $c_{44}^{\left(0\right)}q_{0}$ is replaced by $-\sigma \cdot \omega^{2}$.

As a result, the modified dispersion equation is an implicit function of the phase velocity $v_{p}$ and surface mass density σ, what can be symbolically written as $F\left(v_{p},\;\sigma \right)=0$. The derivative $dv_{p}/d\sigma$ in Equation (35) can be calculated analytically from the modified dispersion equation, using the rules of differentiation of implicit functions. In fact, the differentiation of the modified dispersion equation $F\left(v_{p},\;\sigma \right)=0$ with respect to $v_{p}\;and\;\sigma$ leads to the following relation $\left(dv_{p}/d\sigma \right)\partial F/\partial v_{p}+\left(d\sigma /d\sigma \right)\partial F/\partial \sigma =0$. Consequently, the derivative $dv_{p}/d\sigma$ can be written as:

(36) $$\frac{dv_{p}}{d\sigma }=-\frac{\partial F/\partial \sigma }{\partial F/\partial v_{p}}$$

By virtue of Equations (35) and (36) the coefficient of mass sensitivity $S_{\sigma }^{v_{p}}$ is given by the following explicit formula [19,20]:

(37) $$S_{\sigma }^{v_{p}}=\frac{\omega^{2}\frac{1}{k}\left\{\left(c_{44}^{\left(1\right)}q_{1}\right)+\left(c_{44}^{\left(2\right)}q_{2}\;\right)\cdot tan\left(q_{1}h_{1}\right)\right\}}{\frac{h_{1}}{cos^{2}\left(q_{1}h_{1}\right)}\;\frac{\partial q_{1}}{\partial k}\left\{{\left(c_{44}^{\left(1\right)}q_{1}\right)}^{2}+\left(c_{44}^{\left(2\right)}q_{2}\;\right)\left(\sigma \omega^{2}\right)\right\}\;+tan\left(q_{1}h_{1}\right)\left\{2q_{1}{\left(c_{44}^{\left(1\right)}\right)}^{2}\frac{\partial q_{1}}{\partial k}+c_{44}^{\left(2\right)}\frac{\partial q_{2}\;}{\partial k}\left(\sigma \omega^{2}\right)\right\}+c_{44}^{\left(1\right)}\frac{\partial q_{1}}{\partial k}\cdot \left\{\left(\sigma \omega^{2}\right)-\left(c_{44}^{\left(2\right)}q_{2}\;\right)\right\}-c_{44}^{\left(2\right)}\frac{\partial q_{2}\;}{\partial k}\left(c_{44}^{\left(1\right)}q_{1}\right)}$$

where $h_{1}$ is the thickness of the guiding surface layer, $q_{1}\;and\;q_{2}$ are, respectively, transverse wavenumbers of the Love wave in the guiding surface layer and in the substrate and $\partial q_{1}/\partial k=-k/\sqrt{k_{1}^{2}-k^{2}}$; $\partial q_{2}\;/\partial k=k/\sqrt{k^{2}-k_{2}^{2}}$.

It has to be stressed that Equation (37) is a closed form analytical formula for the mass coefficient of sensitivity $S_{\sigma }^{v_{p}}$, as a function of $\omega ,\;h_{1},\;v_{p},c_{44}^{\left(1\right)},\;\rho_{1},\;c_{44}^{\left(2\right)},\;\rho_{2},\;and\;\sigma$. Equation (37) will be used in the subsequent numerical calculations of the mass sensitivity $S_{\sigma }^{v_{p}}$ for Love surface waves propagating in waveguides composed of a PMMA guiding surface layer deposited on the ST-Quartz substrate (see Section 5.2).

One may wonder what on earth is common between the two waveguide configurations shown in Figure 1 and Figure 2. First waveguide is loaded with a semi-infinite lossy Newtonian liquid (Figure 1) and second with an infinitesimally thin layer of a lossless mass (Figure 2). The first waveguide is lossy with a non-zero amplitude of the Love wave extending from $x_{2}=-\infty$ to $x_{2}=+\infty$ and the second is lossless with the amplitude of the Love wave limited to the positive half-space $x_{2}=\left(0,+\infty \right)$.

Our answer to the above question is the following: the common factor shared by these two seemingly dissimilar waveguide configurations is the fact that in both of them the phase velocity $v_{p}$ of the Love wave is affected by the loading medium, i.e., by a semi-infinite lossy Newtonian liquid or an infinitesimal layer of lossless mass.

Now, we are in a position to go one step further and identify the suspect physical phenomenon, occurring in both waveguides, which is responsible for the phase delay (velocity changes) of the Love wave propagating in both structures. Since loading with a Newtonian liquid gives rise to a non-zero complex Poynting vector $P_{2}^{\left(0\right)}\left(x_{2}=0\right)\neq 0$ at the interface $x_{2}=0$ we postulate that the imaginary part $ImP_{2}^{\left(0\right)}$ of the average power flow $P_{2}^{\left(0\right)}$ in the transverse direction $x_{2}$ in the Newtonian liquid is connected with the phase delay and velocity changes of the Love surface wave. By contrast, the real part $ReP_{2}^{\left(0\right)}$ of the average power flow $P_{2}^{\left(0\right)}$ is connected to losses of the Love wave.

### 4.2. Equivalent Inertial Properties of the Newtonian Liquid Seen by Love Surface Waves

The amplitude of Love surface waves, propagating in waveguides loaded with a Newtonian liquid, decays very rapidly in the Newtonian liquid, as a function of the transverse direction $x_{2}$ (see Figure 1). The penetration depth $\delta_{0}$ of the Love wave into a Newtonian liquid equals $\delta_{0}=1/Req_{0}$, where the real part $Req_{0}=a_{0}$ of the transverse wave number $q_{0}$ of the Love wave in the Newtonian liquid is given by Equation (29). The penetration depth $\delta_{0}$ of the Love wave in the Newtonian liquid is given approximately by [27]:

(38) $$\delta_{0}\approx \sqrt{\frac{2\eta_{0}}{\omega \rho_{0}}}$$

where $\eta_{0}$ is the viscosity and $\rho_{0}$ the density of the Newtonian liquid.

Now, we will answer another question. Can semi-infinite Newtonian liquid, with an exponentially decaying mechanical displacement of the Love wave, be replaced by an equivalent thin layer of a thickness $h^{\prime }$, with a constant mechanical displacement of the Love wave equal to $u_{3}^{\left(0\right)}\left(x_{2}=0\right)$ that provides the same changes in phase velocity $v_{p}$ of the Love wave?

To answer this question we will determine the average power flow $P_{2}^{\left(0\right)}=\int_{-\infty }^{0}P_{2}^{\left(0\right)}\left(x_{2}\right)dx_{2}$ (per unit width along axis $x_{3}$) in the transverse direction $x_{2}$, in the loading Newtonian liquid, where $P_{2}^{\left(0\right)}\left(x_{2}\right)$ is the complex Poynting vector in the Newtonian liquid. Since $P_{2}^{\left(0\right)}\left(x_{2}\right)=P_{2}^{\left(0\right)}\left(x_{2}=0\right)e^{2Re\left(q_{0}\right)x_{2}}$, see Equation (26), the average power flow in the Newtonian liquid equals:

(39) $$P_{2}^{\left(0\right)}=\overset{0}{\underset{-\infty }{\int }}P_{2}^{\left(0\right)}\left(x_{2}\right)dx_{2}=P_{2}^{\left(0\right)}\left(x_{2}=0\right)/2Re\left(q_{0}\right)$$

By definition, the penetration depth $\delta_{0}$ of the Love wave into the loading Newtonian liquid equals $\delta_{0}=1/Req_{0}$. Therefore, we can write that:

(40) $$P_{2}^{\left(0\right)}=P_{2}^{\left(0\right)}\left(x_{2}=0\right)\delta_{0}/2$$

As a result, the sought equivalent thickness $h^{\prime }$ is given by:

(41) $$h^{\prime }=\delta_{0}/2$$

Since, for thin layers of a lossless mass, the surface mass density of the load can be written as $\sigma =\rho_{0}h^{\prime }$, we postulate that the effective mass loading exerted by a semi-infinite Newtonian liquid is equivalent to mass loading of a thin layer of lossless mass of thickness $\delta_{0}/2$ and density $\rho_{0}$. As a result, we can write:

(42) $$\sigma =\rho_{0}\delta_{0}/2=\sqrt{\frac{\rho_{0}\eta_{0}}{2\omega }}$$

where σ given by Equation (42) represents the equivalent surface mass density [$kg/m^{2}$] of a semi-infinite Newtonian liquid loading Love wave waveguide. Note that, the equivalent surface mass density σ depends on density $\rho_{0}$ and viscosity $\eta_{0}$ of the Newtonian liquid as well as on angular frequency ω of the Love wave.

Now, we are able to determine the coefficient of mass sensitivity $S_{\sigma }^{v_{p}}$ of Love wave sensors, loaded with a semi-infinite Newtonian liquid, using Equation (37) with the effective surface mass density σ given by Equation (42).

The coefficient of mass sensitivity $S_{\sigma }^{v_{p}}$ will be plotted in Section 5.2, as a function of frequency f and thickness of the elastic surface layer $h_{1}$.

## 5. Numerical Results

The analytical formulas obtained in this paper will be illustrated by numerical results performed for the Love wave waveguide structure showed in Figure 1 (Section 2) and Figure 2 (Section 4.1) with the waveguide parameters enclosed in Table 1.

The necessary condition for Love surface waves to exist is as follows: $v_{1}<v_{2}$, i.e., the elastic surface layer must be slower than the elastic substrate (see Table 1). In the PMMA elastic surface layer the phase velocity of bulk SH waves $v_{1}=1100\;m/s$ and in the ST-Quartz substrate $v_{2}=5060\;m/s$. Both materials are assumed to be lossless and frequency independent. The piezoelectric effect in the ST-Quartz substrate was neglected except for the phase velocity $v_{2}$ of bulk SH waves in Quartz (see Appendix A for more details). Since the piezoelectric effect in the Quartz is relatively weak $\left(\sim 0.3\%\right)$ the above assumption can be considered as a good first order approximation.

### 5.1. Average Power Flow $P_{2}^{\left(0\right)}$ in the Transverse Direction $x_{2}$ in the Newtonian Liquid

The average power flow $P_{2}^{\left(0\right)}$ (Equation (39)) in the transverse direction $x_{2}$ in the Newtonian liquid is a complex quantity; therefore, it can be written as $P_{2}^{\left(0\right)}=ReP_{2}^{\left(0\right)}+jImP_{2}^{\left(0\right)}$. The active part $ReP_{2}^{\left(0\right)}$ of the average power flow $P_{2}^{\left(0\right)}$ is converted to heat in the Newtonian liquid. The reactive part $ImP_{2}^{\left(0\right)}$ of the average power flow $P_{2}^{\left(0\right)}$ is responsible for storage of the energy in the Newtonian liquid and ultimately leads to phase delay of the Love wave and changes in its phase velocity.

**Figure 3:**
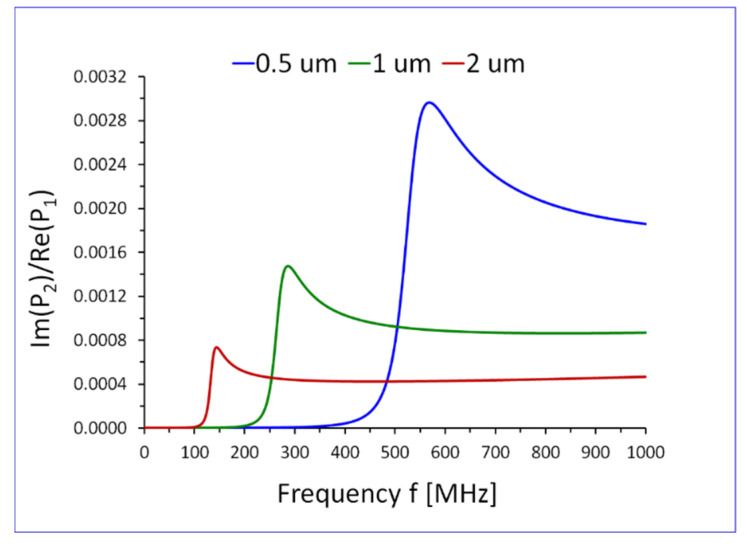
Reactive part $ImP_{2}^{\left(0\right)}$ of the average power flow $P_{2}^{\left(0\right)}$ in the transverse direction $x_{2}$, in the Newtonian liquid, normalized by the active average power flow $Re(P_{1}$) in the direction of propagation $x_{1}$, in the entire waveguide, shown in Figure 1. The plots are drawn as a function of frequency f, for different values of thickness $h_{1}$ of guiding surface layer $h_{1}=0.5,\;1\;\mathrm{and}\;2\;\mu m$. The waveguide is loaded with a Newtonian liquid of density $\rho_{0}=1000\;\mathrm{kg}/m^{3}$ and viscosity $\eta_{0}=1\;\mathrm{mPas}$.

**Figure 4:**
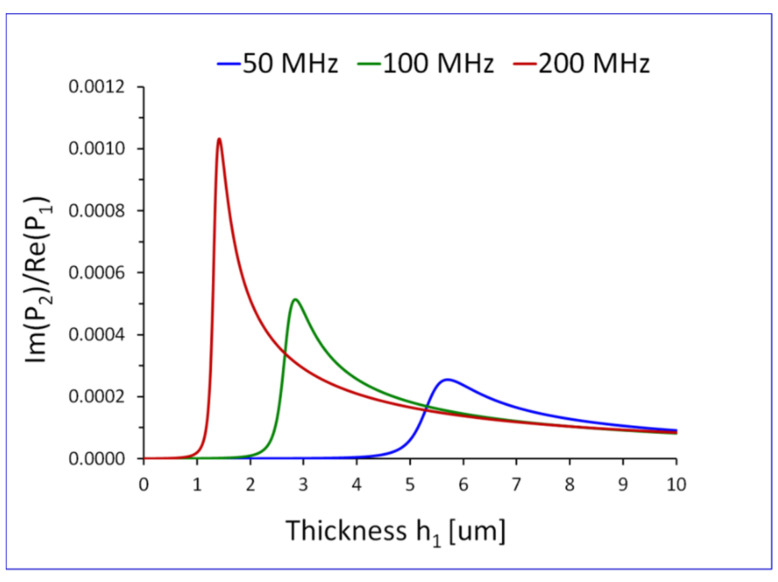
Reactive part $ImP_{2}^{\left(0\right)}$ of the average power flow $P_{2}^{\left(0\right)}$, in the transverse direction $x_{2}$, in the Newtonian liquid, normalized by the active average power flow $Re(P_{1}$) in the direction of propagation $x_{1}$, in the entire waveguide, shown in Figure 1. The plots are drawn as a function of thickness $h_{1}$ of the elastic surface layer, for different values of frequency f of the wave f=50, 100 and 200 MHz. The waveguide is loaded with a Newtonian liquid of density $\rho_{0}=1000\;\mathrm{kg}/m^{3}$ and viscosity $\eta_{0}=1\;\mathrm{mPas}.$.

In Figure 3 and Figure 4 we present the reactive part $ImP_{2}^{\left(0\right)}$ of the average power flow $P_{2}^{\left(0\right)}$ in the Newtonian liquid, as a function of frequency f and thickness of the elastic surface layer $h_{1}$. It is evident that $ImP_{2}^{\left(0\right)}$ exhibits pronounced maxima as a function of f and $h_{1}$. The plots of $ImP_{2}^{\left(0\right)}$ are normalized by the active average power flow $Re(P_{1}$), see Equation (34) in the direction of propagation $x_{1}$, in the entire waveguide, shown in Figure 1.

### 5.2. Coefficient of Mass Sensitivity $S_{\sigma }^{v_{p}}$

By contrast to the average power flow $P_{2}^{\left(0\right)}$, presented in Section 5.1, the coefficient of mass sensitivity $S_{\sigma }^{v_{p}}$ is a real-valued quantity, since by definition (Equation (35)) it is related only to changes in phase velocity of the Love wave.

The coefficient of mass sensitivity $S_{\sigma }^{v_{p}}$ of Love wave sensors, loaded with a semi-infinite Newtonian liquid, was determined using Equation (37) with the effective surface mass density σ of the Newtonian liquid given by Equation (42).

**Figure 5:**
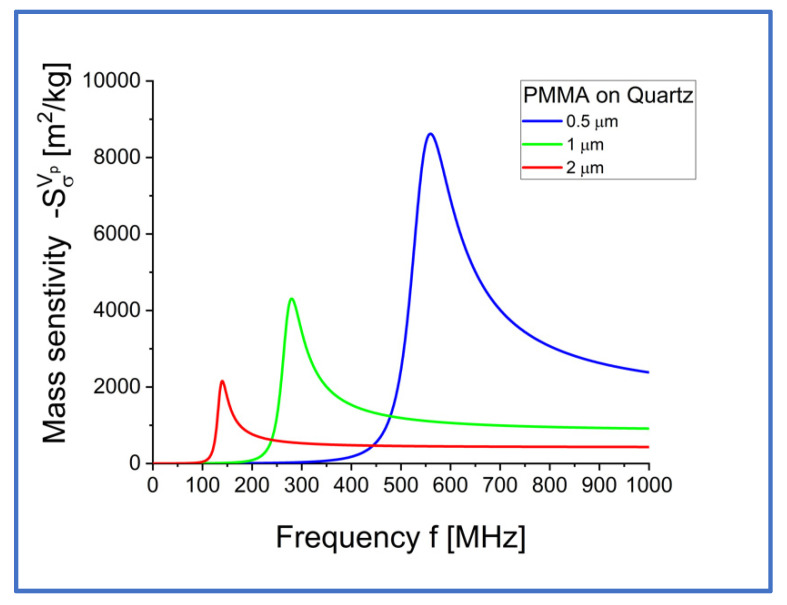
Coefficient of mass sensitivity $S_{\sigma }^{v_{p}}\;\left[m^{2}/\mathrm{kg}\right]$ for Love surface waves propagating in waveguides loaded with a semi-infinite Newtonian liquid with an equivalent surface mass density $\sigma =\rho_{0}\delta_{0}/2$, as a function of frequency f, for different values of thickness $h_{1}$ of the elastic surface layer ($h_{1}=0.5,\;1\;\mathrm{and}\;2\;\mu m)$.

**Figure 6:**
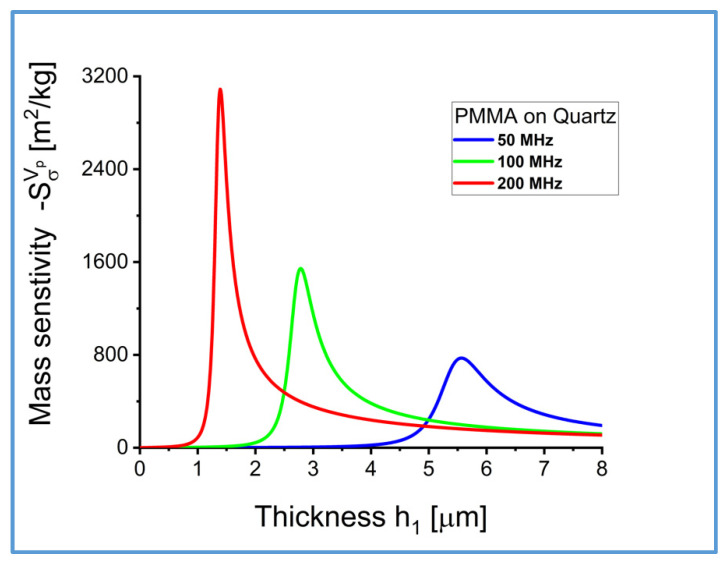
Coefficient of mass sensitivity $S_{\sigma }^{v_{p}}\;\left[m^{2}/\mathrm{kg}\right]$ for Love surface waves propagating in waveguides loaded with a semi-infinite Newtonian liquid with an equivalent surface mass density $\sigma =\rho_{0}\delta_{0}/2$, as a function of thickness $h_{1}$ of the elastic surface layer, for different values of frequency f=50, 100 and 200 MHz.

The coefficient of mass sensitivity $S_{\sigma }^{v_{p}}$ is plotted in Figure 5 and Figure 6, as a function of frequency f and thickness of the elastic surface layer $h_{1}$.

## 6. Discussion

In this paper, we investigated two phenomena occurring in Love wave waveguides loaded with a semi-infinite Newtonian liquid of a viscosity $\eta_{0}$ and density $\rho_{0}$. First, the average power flow $P_{2}=\int_{-\infty }^{\infty }P_{2}\left(x_{2}\right)dx_{2}$, in the transverse direction $x_{2}$, and the corresponding complex Poynting vector $P_{2}^{\left(0\right)}\left(x_{2}=0\right)$, evaluated at the interface $x_{2}=0$, between the waveguide and the Newtonian liquid. Second, the change $∆v_{p}/v_{p}$ in phase velocity of the Love wave, represented by the mass coefficient of sensitivity $S_{\sigma }^{v_{p}}$.

At first glance, these two phenomena may seem to be completely unrelated. However, in this paper we showed that the first phenomenon provides physical basis for the second one. In fact, if the average reactive power flow across the interface between the waveguide and the Newtonian liquid, represented by the imaginary part of the Poynting vector $ImP_{2}^{\left(0\right)}\left(x_{2}=0\right)=0$ vanishes then the phase velocity of the Love wave remains unchanged and consequently $S_{\sigma }^{v_{p}}=0$.

It is commonly accepted that the attenuation of the Love wave is caused by viscous properties of the Newtonian liquid, represented by its viscosity $\eta_{0}$. On the other hand, the change in phase velocity of the Love wave can be attributed to inertial properties of the Newtonian liquid, represented by its density $\rho_{0}$. To be more specific, both phenomena, i.e., the attenuation and changes in phase velocity of the Love wave depend on both parameters of the Newtonian liquid, namely on $\eta_{0}$ and $\rho_{0}$ in a quite involved manner (see Equations (29) and (30)).

By definition, the mass coefficient of sensitivity $S_{\sigma }^{v_{p}}\approx \Delta v_{p}/\Delta \sigma /v_{p}$ quantifies changes in phase velocity of the Love wave due to loading with an infinitesimally thin layer of a lossless mass. However, in this paper we showed that mass loading with a semi-infinite Newtonian liquid is equivalent to loading with a thin layer of mass of density $\rho_{0}$ and thickness $\delta_{0}/2$, where $\delta_{0}$ is the penetration depth of the Love wave into the Newtonian liquid. As a result, we are able to determine the mass coefficient of sensitivity $S_{\sigma }^{v_{p}}$ for a semi-infinite Newtonian liquid loading the waveguide assuming that the effective surface mass density of a semi-infinite Newtonian liquid equals $\Delta \sigma =\rho_{0}\delta_{0}/2\;\left[kg/m^{2}\right]$.

To reduce the magnitude of numbers of on Y-axis in Figure 3 and Figure 4, the reactive part $ImP_{2}$ of the average power flow $P_{2}$ in the Newtonian liquid in the transverse direction $x_{2}$, was normalized by the active part $ReP_{1}$ of the average power flow $P_{1}$ in the direction of propagation $x_{1}$.

## 7. Conclusions

Based on the results of the theoretical analysis and the corresponding numerical calculations, obtained in this paper, we can draw the following conclusions:

1. The non-zero complex Poynting vector $P_{2}^{\left(0\right)}\left(x_{2}\right)$ in the transverse direction $x_{2}$, evaluated at the interface $x_{2}=0$, between the loading Newtonian liquid and the elastic surface layer of the waveguide, represents extra active and reactive power fluxes occurring in the Love wave waveguide loaded with a Newtonian liquid.
2. The active part $ReP_{2}$ of the average power flow $P_{2}$ in the transverse direction $x_{2}$ feeds viscous losses in the Newtonian liquid and therefore is connected to attenuation of the Love wave.
3. The reactive part $ImP_{2}$ of the average power flow $P_{2}$ in the transverse direction $x_{2}$ in the Newtonian liquid delays propagation of the Love wave in the direction $x_{1}$ and therefore is connected with changes of the phase velocity of the Love wave and in turn with the coefficient of mass sensitivity $S_{\sigma }^{v_{p}}$ of the sensor.
4. The changes in phase velocity $\Delta v_{p}/v_{p}$ of the Love wave, propagating in waveguides loaded with a semi-infinite Newtonian liquid of a viscosity $\eta_{0}$ and density $\rho_{0}$ and the changes in phase velocity in waveguides loaded by a lossless layer of mass with a density $\rho_{0}$ and thickness $\delta_{0}/2$, are the same, where $\delta_{0}$ is the penetration depth of the Love wave into the Newtonian liquid.
5. The maxima of the coefficient of mass sensitivity $S_{\sigma }^{v_{p}}$ and the maxima of the reactive part of the average power flow in the transverse direction $x_{2}$ occur virtually for the same values of the frequency f and thickness $h_{1}$ of the elastic surface layer.

The results of the theoretical analysis and numerical calculations presented in this paper provide new physical insight for the coefficient of mass sensitivity $S_{\sigma }^{v_{p}}$ of Love wave sensors. The relation between the extra reactive power flow across the interface $x_{2}=0$, separating the loading Newtonian liquid and the elastic surface layer of the waveguide, and the mass sensitivity $S_{\sigma }^{v_{p}}$ can be a basis of optimal design of ultrasonic Love wave sensors.

## Figures and Tables

**Table 1:** Material and geometrical parameters of the Love wave waveguide.

Waveguide Component	Material Type	Thickness [μm]	Density $\left[kg/m^{3}\right]$	Shear Modulus [GPa]	Phase Velocity of SH Bulk Waves [m/s]
Elastic surface layer	PMMA	$h_{1}=0-10$	$\rho_{1}=1180$	$c_{44}^{\left(1\right)}=1.43$	$v_{1}=1100$
Elastic substrate	ST-cut Quartz	semi-infinite	$\rho_{2}=2650$	$c_{44}^{\left(2\right)}=67.85$	$v_{2}=5060$

## Data Availability

Not applicable.

## Appendix A

### Quartz Material as a Substrate in Love Wave Sensors

Quartz crystal belongs to 32 class of the trigonal system of symmetry (see page 207 in [3]) and in general have 5 independent elastic constants $c_{11},\;c_{12},\;c_{13},\;c_{14},\;c_{44}$ and $c_{66}=\left(c_{11}-c_{12}\right)/2)$, 2 piezoelectric constants $e_{11},\;e_{14}$ and 2 dielectric constants $\varepsilon_{11},\;\varepsilon_{33}$. However, the phase velocity $v_{2}=\sqrt{c_{44}^{\left(2\right)}/\rho_{2}}$ of pure shear SH volume waves in Quartz, polarized along the X crystallographic axis and propagating in directions located in the Y-Z plane of symmetry, depends only on one shear modulus of elasticity $c_{44}^{\left(2\right)}$ given by the following Formula (A1):

(A1) $$c_{44}^{\left(2\right)}=c_{66}sin^{2}\beta +c_{44}cos^{2}\beta -c_{14}sin\left(2\beta \right)+\frac{{\left[e_{11}sin^{2}\beta -\left(e_{14}/2\right)sin\left(2\beta \right)\right]}^{2}}{\varepsilon_{11}sin^{2}\beta +\varepsilon_{33}cos^{2}\beta }$$

where the angle $\beta =42.5^{{}^{\circ}}$ for the ST-Quartz crystal. Substituting the appropriate values for $c_{66},\;c_{44},\;c_{14},\;e_{11},e_{14},\;\varepsilon_{11},\;\varepsilon_{33}\;(\mathrm{see}\;\mathrm{pages}\;148\;\mathrm{and}\;164\;\mathrm{in}$ [3]) and $\rho_{2}=2650\;\mathrm{kg}/m^{3}$ one obtains $v_{2}=\sqrt{c_{44}^{\left(2\right)}/\rho_{2}}=5060\;m/s$. Therefore, this value of the phase velocity of bulk SH waves in ST-Quartz was used in calculations presented in this paper.
